# Molecular detection of *Ehrlichia chaffeensis* in marsh deer (Blastocerus dichotomus) and their parasitic *Amblyomma triste* ticks in Argentina suggests a local transmission cycle

**DOI:** 10.1186/s13071-025-07211-1

**Published:** 2026-01-02

**Authors:** Eliana Carolina Guillemi, María Marcela Orozco, Iara Figini, Paula Blanco, Marisa Diana Farber

**Affiliations:** 1 Instituto de Agrobiotecnología y Biología Molecular (IABIMO), INTA-CONICET, Hurlingham, B1686LQF Argentina; 2 Facultad de Ciencias Exactas y Naturales, Departamento de Ecología, Genética y Evolución, Universidad de Buenos Aires, Buenos Aires, Argentina; 3 Instituto de Ecología, Genética y Evolución de Buenos Aires (IEGEBA), Consejo Nacional de Investigaciones Científicas y Técnicas, Buenos Aires, Argentina

**Keywords:** *Ehrlichia chaffeensis*, Marsh deer, *Amblyomma triste*, Salivary glands, South America

## Abstract

**Background:**

Since the first finding of *Ehrlichia chaffeensis* in the Argentinian marsh deer populations in 2018, we have conducted ongoing surveillance efforts to better understand the ecological and epidemiological dynamics of this zoonotic tick-borne pathogen in the region.

**Methods:**

Over a period of 7 years (2018–2024) we analyzed blood and tissue samples from 40 marsh deer (*Blastocerus dichotomus*) and 13 parasitic *Amblyomma triste* ticks in the Paraná River Delta, Argentina.

**Results:**

We identified *E. chaffeensis* DNA in two deer blood samples and in the salivary glands of three *A. triste* ticks parasitizing one of those deer. The approach used for *E. chaffeensis* detection in the tick sample ensured that the identified DNA came from an active tick infection, not residual host blood in the gut. After sequencing generic genes (*16S ribosomal RNA [rRNA], 23S rRNA* and *groEl*) and the specific *E. chaffeensis* variable-length polymerase chain reaction target (VLPT) molecular marker, we identified a genotype previously reported in Argentina.

**Conclusions:**

This first-time contemporary detection of zoonotic *E. chaffeensis* in both a wild vertebrate host and its parasitic generalist ticks in a natural setting, provides direct evidence of a putative transmission cycle in Argentina, highlighting the need of implementing multidisciplinary surveillance systems.

**Graphical Abstract:**

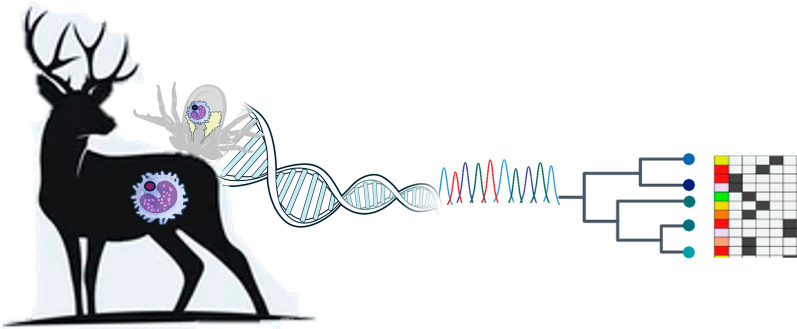

**Electronic Supplementary Material:**

The online version contains electronic supplementary material available at 10.1186/s13071-025-07211-1.

## Background

Emerging and re-emerging vector-borne diseases are among the major public health concerns around the world [1]. These diseases impact both human and animal populations, with increasing incidence driven by factors including climate change, habitat alteration, and urban expansion [2]. In response to this growing threat, the World Health Organization (WHO) launched the Global Vector Control Response 2017–2030 (GVCR), aiming to strengthen vector surveillance and promote integrated control strategies. In this context, the study of infectious agents at the human-animal-environment interface is a critical aspect of such efforts.

*Ehrlichia chaffeensis* (Anaplasmataceae family), is a Gram-negative obligate intracellular bacterium transmitted by hard ticks mainly among humans, deer, and canids [3]. When infecting humans, this tick-borne pathogen is responsible for a life-threatening disease known as human monocytic ehrlichiosis (HME) [4]. This flu-like illness usually requires hospitalization in more than half of the cases [5]. In the USA the HME is maintained in an epidemiological cycle involving white-tailed deer (*Odocoileus virginianus*) as the main vertebrate reservoir and the lone star tick (*Amblyomma americanum*) as its principal vector. In South America, *E. chaffeensis* has been detected in ticks (*Amblyomma parvum* and *Riphicephalus microplus*) and cervids (*Blastocerus dichotomus* and *Mazama gouazoubira*) through specific DNA target fragments [6–9]. Since HME is a flu-like neglected and underdiagnosed disease, few reports and confirmed cases are found in the bibliography. In the context of a cluster of tick-borne disease clinical cases in northern Argentina, Ripoll et al. [10] demonstrated for the first time in Argentina human serologic responses to *E. chaffeensis* or an antigenically related *Ehrlichia* species. Later, in 2016, Halac [11] described a case report of a 12-year-old child who, after being bitten by ticks, suffered unspecific symptoms and tested positive for *E. chaffeensis* or a related species, through serology (immunofluorescence assay [IFA]) and molecular detection (*Ehrlichia* sp.). Other South American countries have reported positive serosurves for *Ehrlichia sp.*, including Brazil [12–15], Chile [16], and Peru [17]. Additionally, novel *Ehrlichia* species, different from but closely related to the *E. chaffeensis*, have been detected through molecular methods in *Amblyomma* species ticks in Argentina [18–20].

Even with these scarce reports, to the best of our knowledge, no studies have shown any evidence of a connection between a vector and vertebrate reservoirs for maintaining *E. chaffeensis* in these environments. In Argentina, our group reported the molecular detection of *E. chaffeensis* in *A. parvum* ticks from semiarid southern Chaco [7], and later in blood samples collected from marsh deer (*Blastocerus dichotomus*) in the alluvial plain of the Paraná River [9]. In this last study, we also detected *E. chaffeensis* in fully engorged *R. microplus* ticks collected from the same infected marsh deer, though we could not infer a vector role, as the blood meal was included in the analyzed specimens. Particularly, the marsh deer is a threatened native cervid from South America, primarily inhabiting wetland ecosystems across Argentina, Bolivia, Peru, Brazil, Uruguay, and Paraguay, with populations undergoing a marked decline due to multiple anthropogenic pressures, including habitat loss and degradation, illegal hunting, and diseases [21, 22]. Since this first finding of *E. chaffeensis* in the Argentinian marsh deer populations, we have conducted ongoing surveillance efforts to better understand the ecological and epidemiological dynamics of this zoonotic tick-borne pathogen in the region [23, 24].

Among the tick species parasitizing marsh deer, *Amblyomma triste* is one of the most frequent and widely distributed in the Southern Cone of South America. This three-host tick parasitizes a broad spectrum of wild and domestic mammals across wetland and riparian habitats [25]. Because *Ehrlichia* spp. are not transmitted transovarially, at least one of the developmental stages of the tick (larva, nymph, adult) must feed on competent reservoirs to maintain the bacterium in nature. Understanding the ecology, host associations, and seasonal dynamics of *A. triste* in the Paraná River Delta is therefore critical to interpret its potential involvement in *E. chaffeensis* transmission cycles. In the present study we analyzed the occurrence of *E. chaffeensis* in marsh deer from wetlands along the Paraná River, testing both deer samples and their *Amblyomma triste* parasitizing ticks.

## Methods

### Study area and sample collection

As part of a participatory surveillance, we studied morbidity and mortality events in the marsh deer population at the Paraná River Delta region in the Buenos Aires province of Argentina (located between 60° 39′ W, 32° 60′ S and 58° 30′ W, 34° 30′ S) [23, 24]. Over a period of 7 years (2018–2024) the members of the collaborative network remained alert for any reports of disease or mortality events affecting marsh deer populations. Upon notification of such events, a field team was mobilized to the affected site to conduct field investigations. Complete necropsies were performed on succumbed marsh deer, and biological samples were systematically collected. When possible, each deer was sexed and assigned to an age category (fawn, yearling, or adult) based on tooth eruption and wear patterns [26]. The body condition of the deer was determined using the Body Condition Scoring Chart [27]. Tick examination was conducted by visual inspection, and in those cases where the deer were parasitized, tick burden was estimated based on the method described by Orozco et al. [23]. Ticks were collected from deer using fine-point tweezers and preserved in tubes containing 70% ethanol until further identification. Full blood samples were collected from the heart of recently succumbed animals (within the hour) via cardiac puncture (2 mL). Aliquots were stored at 4 °C in tubes containing Ethylenediaminetetraacetic acid (EDTA), while additional aliquots were frozen at −20 °C and −80 °C without additives. For those dead deer that presented signs of autolysis, we sampled the ear rim by performing a tissue incision (1 × 1 cm) using a scalpel and conserved this at −20 °C until further analysis.

The study adhered to the ethical and biosafety standards of the Faculty of Exact and Natural Sciences, University of Buenos Aires, with approval from the Institutional Animal Care and Use Committee (Protocol N° 2021/164). Capture and transit permits were obtained from the National Parks Administration (NEA 513 Rnv 1).

### Ticks taxonomic identification and processing

Ticks were identified under a stereoscopic magnifier (SMZ-2 T Nikon, Sendai, Natori, Japón) using taxonomic keys [28]. After identification (Electronic Supplementary Material [ESM] Fig. S1A–B), ticks were subjected to dissection to recover the salivary glands (SG). The specimens were first rinsed in 1 mL of sterile 1× phosphate-buffered saline (PBS; Sigma-Aldrich, Saint Louis, MO, USA), and then, the ventral cuticle was released using a scalpel blade (ESM Fig. S1C). The SG were identified, removed from the tick body (ESM Fig. 1D), washed with sterile 1× PBS, and conserved at −20 °C until further processing [29, 30]. To identify the contaminants potentially incorporated during sample processing [31], we systematically collected an aliquot of 1× PBS from each Petri dish after dissection (dissection controls).

### Detection of *E. chaffeensis* from deer and tick samples

DNA extraction from deer blood and ear samples was performed using the DNeasy Blood and Tissue Mini Kit (QIAGEN. Hilden, Germany) following the protocols recommended by the manufacturer. DNA from the dissected tick’s SG was extracted using the Nucleospin Tissue Kit (Macherey–Nagel. Düren, Germany) following the manufacturer’s instructions. In all cases DNA was stored at −20 °C until further use.

Detection of *E. chaffeensis* DNA from deer and tick samples was conducted by amplifying family- and species-specific gene targets. We first screened all samples by targeting the *16S rRNA*, *23S rRNA* and *groEl* genes common to Anaplasmataceae [32–34]. For those positive samples we further used two sets of primers specific to *E. chaffeensis*, one of them targeting a fragment of the *16S rRNA gene* [35] and the other targeting the variable-length polymerase chain reaction (PCR) target (VLPT) region [36]. This last protocol was also used for *E. chaffeensis* genotyping by sequencing the PCR fragment amplified from this size variation gene [36]. All PCR reactions were performed in 50 µL reaction mixture (0.2 mM of each deoxyribonucleotide triphosphate, 1.25 U of GoTaq DNA polymerase [Promega Madison, WI, USA], 10 μL of 5× PCR buffer). The primer final concentration was 0.4 μmol of each oligonucleotide except for the *groEl* gene reaction mixture. In this last PCR reaction, as primers are degenerate, we optimized final concentrations to 0.56 μmol (oligo_1236) and 0.8 μmol (oligo_643). Positive (Arkansas reference strain) and negative (pure water) controls were included in each PCR reaction. For visualization of the amplicons, 5 μL of mixture were visualized through electrophoresis in 1% agarose gel stained with ethidium bromide. A molecular size marker (1 Kb Plus DNA Ladder, Invitrogen) was used to determine PCR product size. For positive reactions, the remaining 45 μL of mixture were purified using a commercial kit (Monarch Spin PCR & DNA Cleanup Kit, NEB, Whitby, ON, Canada) according to the manufacturer’s instructions.

Both strands from *16S rRNA* (Anaplasmataceae), *23S rRNA, groEL* and VLPT fragments were sequenced with a Big Dye Terminator v3.1 kit from Applied Biosystems and analyzed on an ABI 3130XL genetic analyzer from the same supplier (Unidad de Genómica y Bioinformática, IABIMO INTA-CONICET). For each PCR fragment, forward and reverse chromatograms (ab1 files) were used for assembling, and the outcome contig (FASTA file) was used for further phylogenetic and characterization analysis.

### Phylogenetic analysis and molecular characterization

Evolutionary analyses were conducted in MEGA6 [37]. Sequences from the *16S rRNA*, *23S rRNA*, and *groEl* genes (three gene targets common to Anaplasmataceae) were used for constructing phylogenetic trees. The evolutionary relation among sequences was inferred by using the maximum likelihood method based on the Kimura two-parameter model [38]. A discrete gamma distribution was used to model evolutionary rate differences among sites. Node robustness was assessed with 1000 bootstrap replicates. Apart from the sequences obtained in this study, we also included sequences from reference species in the family Anaplasmataceae and a sequence from *Rickettsia* species as outgroup. For tree visualization and labels edition, we employed the iTOL.v7 program [39].

For molecular characterization through VLPT target, we first employed an online bioinformatic tool available at Expasy website [40] for the translation of the nucleotide VLPT sequences into proteins. After translation, we searched for tandemly repeated amino acid sequences in each sample. The number of repeats and amino acid sequence type defined the genotypes present in each sample.

## Results

### Ticks taxonomic identification

All parasitic ticks studied were taxonomically identified as *Amblyomma triste*. We collected and analyzed 13 ticks; these specimens were all adults, corresponding to females (*n* = 2) and males (*n* = 11). These ticks were collected from two deer, CP_499 and CP_508, with low and medium tick burdens, respectively (Table 1).

**Table 1:** Summary of ticks collected and analyzed in the present study from deer hosts CP_499 and CP_508

Tick source	*Amblyomma triste*	
	Adult female	Adult male
CP_499	2	3
CP_508	—	8
Total	2	11

CP_499 and CP_508 are the deer hosts’ tags

### Detection of *E. chaffeensis* from deer and tick samples

We assessed the *E. chaffeensis* presence in blood or ear samples from *B. dichotomus* (*n* = 40) as well as in SG from parasitic ticks (*n* = 13) collected from two succumbed deer. From the 40 tested deer samples, 11 were positive to the generic Anaplasmataceae reactions (*16S rRNA*, *23S rRNA*, and *groEl* genes), and 2 of them (CP_506 and CP_508) also tested positive for the *E. chaffeensis*-specific PCR reactions (*16S rRNA* and VLPT) (Table 2). Both *E. chaffeensis*–positive marsh deer were juveniles, with CP506 exhibiting a regular body condition and CP508 showing a poor condition. Additionally, three male *A. triste* ticks (from a total of eight collected) from a positive deer (CP_508) tested positive for the Anaplasmataceae generic (*16S rRNA*, *23S rRNA*, and *groEl*), and *E. chaffeensis*-specific (*16S rRNA* and VLPT) reactions. The other deer hosting *A. triste* ticks (CP_499) and their SG tested negative to the PCR reactions. Those samples that tested positive for Anaplasmataceae PCR reactions but were negative for *E. chaffeensis* protocols (*n* = 9) were purified and sequenced for identification. These samples corresponded to *Anaplasma* species (data not shown). All tick dissection controls tested negative to the Anaplasmataceae *16S rRNA* protocol.

**Table 2:** Summary of the gene targets studied for each sample

Sample	Gene targets				
	*16S rRNA* (Anaplasmataceae)	*23S rRNA* (Anaplasmataceae)	*groEl* (Anaplasmataceae)	*16S rRNA* (*E. chaffeensis*)	VLPT (*E. chaffeensis*)
CP_506	Sequenced (PV785953)	Sequenced (PV786002)	Sequenced (PV861766)	PCR positive	Sequenced (PV791126)
CP_508	Sequenced (PV785954)	Sequenced (PV786003)	Sequenced (PV868190)	PCR positive	Sequenced (PV791127)
SG.At_508	Sequenced (PV785955)	Sequenced (PV786004)	Sequenced (PV861764)	PCR positive	Sequenced (PV861765)

CP: marsh deer samples; SG.At: salivary glands from *Amblyomma triste* tick. For those gene targets that were sequenced, accession numbers are mentioned in brackets

### *Ehrlichia chaffeensis* population variability analysis

We used the obtained nucleotide sequences from the *16S rRNA* (Anaplasmataceae), *23S rRNA*, *groEL*, and VLPT fragments from both deer samples (CP_506 and CP_508) and from one of the positive ticks (SG.At_508) for further molecular characterization. All retrieved sequences have been deposited in GenBank (Table 2).

**Fig. 1:**
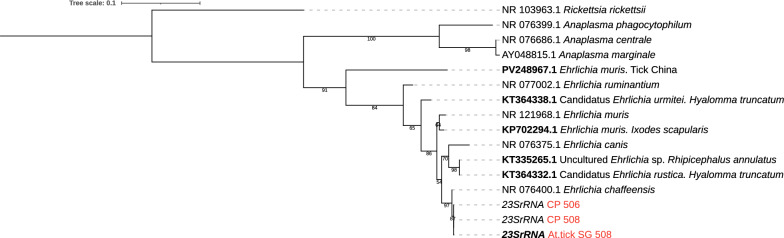
Phylogeny for *23S rRNA* gene based on 458 bp. Maximum likelihood tree. Substitution model: Kimura two-parameter model with discrete Gamma distribution. Phylogeny was tested with 1000 replications of bootstrap. Sequences corresponding to the samples under study (CP_506, CP_508, and SG.At_508) are labeled in red (bold font was used for the tick sample). Accession numbers from other *Ehrlichia* sp. found in tick samples are labeled with bold font. iTOL.v7 program was used for tree visualization

**Fig. 2:**
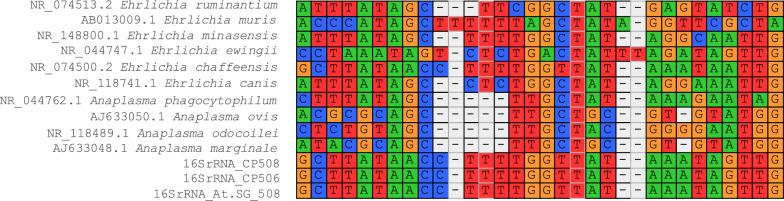
Alignment of the hypervariable V1 region in the *16S rRNA* gene from reference *Ehrlichia* and *Anaplasma* strains and those amplified in the present study (16SrRNA_CP506, 16SrRNA_CP508, and 16SrRNA_At.SG_508). Image obtained with the alignment tool from the Mega 6.06 program

We first performed a local alignment using the BLAST bioinformatic tool available at the National Center for Biotechnology Information (NCBI) website (www.ncbi.nlm.nih.gov). Using the longest sequences obtained for each gene in our samples, the results of this alignment for the analyzed fragments retrieved the following sequences as first hits: for the *16S rRNA* gene, 99.56% identity (CP_508 sequence) with *E. chaffeensis* reference strains (CP007476.1, CP007473.1, CP007477.1, CP007475.1, CP007478.1, CP007480.1, CP000236.1, CP007479.1); for the *23S rRNA* gene, 99.81% identity (CP_508 sequence) with *E. chaffeensis* reference strains (CP007476.1, CP007473.1, AF416765.1, CP007475.1, CP007477.1, CP007478.1, CP007479.1, CP007480.1); and for the *groEl* gene, we found 99.83% identity (At.SG_508) with a recently proposed *Ehrlichia* sp. strain San Luis (KY425415.1) from *A. tigrinum* ticks and 99.16% identity with an *E. chaffeensis* identified in *Blastocerus dichotomus* from Brazil (JQ085941.1). To confirm the specific assignment and intraspecific variability, nucleotide sequences from *16S rRNA, 23S rRNA* and *groEl* gene fragments were used for phylogenetic analysis. The tree with the highest log likelihood (−1655,2686) corresponding to a fragment of the *23S rRNA* gene is shown in Fig. 1. We included reference sequences from the Anaplasmataceae family (AY048815.1, NR_076399.1, NR_076686.1, NR_077002.1, NR_076400.1, NR_076375.1, NR_121968.1) and a reference sequence from *Rickettsia rickettsii* (NR_103963.1) as an outgroup. Phylogenetic trees for *16S rRNA* and *groEl* genes retrieved equivalent evolutionary associations (ESM). However, through the alignment of the *16S rRNA* sequences with those from reference Anaplasmataceae species, we identified that the hypervariable V1 region in our samples corresponded to *E. chaffeensis* (Fig. 2).

For studying the subspecific variability of the VLPT target gene, the nucleotide sequences obtained were translated into protein sequences to further detect tandemly repeated amino acid blocks. VLPT sequences from both marsh deer samples (CP_506 and CP_508) and from the studied tick SG (At.SG_508) yielded identical amino acid sequences and thus the same tandem repeat profile. The VLPT tandem repeat profile detected for the three samples was also identical to the one previously identified for marsh deer samples in Argentina in previous years and shared some repeat units with VLPT genotypes detected in *A. parvum* ticks from the same country (Table 3).

**Table 3:**
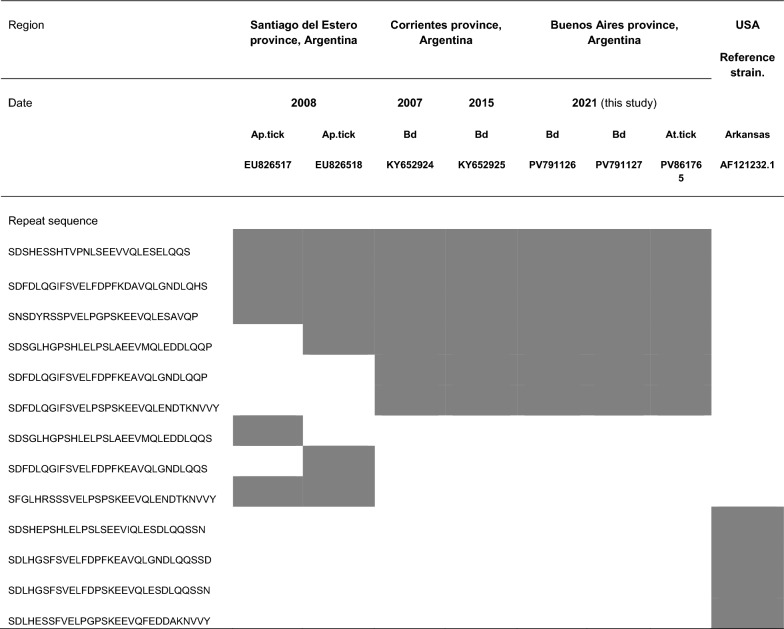
Amino acid sequences for VLPT tandem repeats and profiles for previously reported *E. chaffeensis* in Argentina (in Santiago del Estero and Corrientes provinces), samples in the present study (Buenos Aires province) and the *E. chaffeensis* reference strain Arkansas (used as positive control)

Ap.tick: *Amblyomma parvum* tick, Bd: *Blastocerus dichotomus*, At.tick: *Amblyomma triste* tick

## Discussion

In the present study, we report the molecular identification of *E. chaffeensis* DNA in marsh deer blood samples and in salivary glands from its parasitic *A. triste* ticks. Through molecular detection we were able to amplify fragments from different target genes (*16S rRNA*, *23S rRNA*, *groEL*, and VLPT) in blood samples from two deer and in salivary glands from three *A. triste* ticks. Additionally, we analyzed the sequences corresponding to these four genes from both deer samples (CP_506 and CP_508) and from one of the positive tick’s salivary glands (SG.At_508).

Through the phylogenetic analysis using reference sequences from the Anaplasmataceae family, we observed a clear association between our samples and the reference *E. chaffeensis* sequences, both for non-coding *16S rRNA* and *23S rRNA* genes and the protein coding *groEl* gene. Also, we incorporated into the *groEl* phylogenetic analysis, available sequences from novel proposed *Ehrlichia* species. These two strains, named *Ehrlichia* sp. strain San Luis [18] and *Ehrlichia* sp. strain delta [41], have been detected in *A. tigrinum* and *A. triste* ticks, respectively. One of them (*Ehrlichia* sp. strain San Luis) conformed a clade together with *E. chaffeensis* reference strain, an *E. chaffeensis* sequence retrieved from a *B. dichotomus* in Brazil [8] and our samples. We could not compare our *16S rRNA* nor *23S rRNA* sequences with those from *Ehrlichia* sp. strain delta and *Ehrlichia* sp. strain San Luis because sequences were not obtained for these strains or not available at GenBank. After species identification, further analysis enabled us to genotypically characterize the strains found. The VLPT gene encodes the surface-expressed TRP32 antigen. This protein has variable numbers of amino acid tandem repeats that are useful for *E. chaffeensis* genotypes identification [36, 42, 43]. In the present study we were able to amplify and sequence the corresponding VLPT fragments from both marsh deer samples (CP_506 and CP_508) and from the *A. triste* salivary glands (SG.At_508). The unique VLPT genotype identified for these samples was identical to previous genotypes detected in marsh deer from Argentina [9] and shared repeat blocks with previously reported *E. chaffeensis* in *A. parvum* tick samples from the same country [7]. The first identification of *E. chaffeensis* in Argentina in those *A. parvum* ticks took place 1000 km away from the area in which, years later, the pathogen was detected for the first time in marsh deer. It is worth noting that, while *A. parvum* ticks were sampled from a semiarid area, marsh deer and the *A. triste* ticks in the present study inhabit wetland areas thus highlighting the ubiquity of these findings despite the low detection frequency of *E. chaffeensis* in this country. The recurrence of these genotypes over a period of 17 years in Argentina suggests the circulation of a stable *E. chaffeensis* genotype in the country, different from the genotype corresponding to the North American Arkansas reference strain, originally described in the USA [44].

The identification of *E. chaffeensis* in the *A. triste* salivary glands, represents relevant information regarding the epidemiology of ehrlichiosis in Argentina. Since salivary glands and saliva play an important role in transmission of most tick-borne pathogens to the vertebrate host [45–47], the detection of *E. chaffeensis* in salivary glands suggests that *A. triste* ticks may be responsible for the pathogen transmission to marsh deer. This finding provides key evidence of a link that connects *E. chaffeensis*-positive marsh deer with a tick transmission cycle in the Argentinian wetlands, even though further studies including microscopy or culture among others, are needed to deepen the understanding of *E. chaffeensis* replication dynamics in this tick species. In a previous study we identified, for the first time in Argentina, *E. chaffeensis* in blood from marsh deer [9]. In that study, we also detected *E. chaffeensis* DNA in *Rhipicephalus microplus* ticks collected from positive deer, but due to methodological issues, we could not confirm that those ticks were actually infected and not harboring *E. chaffeensis* in their blood meal. In the present work we improved our methodological approach and studied the tick samples at an organ scale, thus analyzing individual salivary glands. This methodology guarantees that the detection of *E. chaffeensis* DNA in the tick sample corresponds to the presence of the *Ehrlichia* in the arthropod and not to an infected blood meal residue from the tick gut. The incorporation of dissection negative controls validated these PCR results.

From an ecological perspective, the results in the present study deepen our understanding of the pathogen’s natural cycle within a biodiverse and dynamic wetland ecosystem. The Paraná River Delta is not only a critical habitat for marsh deer, a vulnerable and declining species, but also a mosaic of ecological interfaces where human activity (e.g., tourism, livestock grazing, land conversion) increasingly overlaps with wildlife. *Amblyomma triste* is a tick species strongly associated with wetlands and environment prone to flooding in Argentina, Brazil, and Uruguay [28]. While primarily associated with marsh deer, adult stages of *A. triste* also parasitize a wide range of wild and domestic mammals, including humans [25, 48, 49], highlighting their potential as bridge vectors for zoonotic transmission. Additionally, small and medium-size rodents of the families *Cricetidae* and *Caviidae* are the main hosts for immature stages of *A. triste* [25, 50] and could be part of a potential enzootic cycle of *E. chaffeensis* in the area. Recently, an *Ehrlichia* species closely related to *E. chaffeensis* has been detected in an adult questing *A. triste* in the Paraná River Delta [41], suggesting a potential role of this tick in the transmission of *Ehrlichia* agents. Since *Ehrlichia* species circulate among tick stages through transtadial transmission (not transovarially), further studies in the area should focus on the identification of *Ehrlichia* species in immature stages of *A. triste* and their hosts in order to deepen the understanding of the whole enzootic transmission cycle. In the present work, the simultaneous detection of *E. chaffeensis* in both a wild vertebrate host (*Blastocerus dichotomus*) and its parasitic adult *A. triste* ticks in a natural setting brings new evidence regarding the plausible role of *A. triste* in the transmission of *E. chaffeensis*.

Taking into consideration the previous records of seroreaction to *E. chaffeensis* in humans in the country [10], the report of a clinical case [11], and the neglected and underdiagnosed condition of this pathogen in our country, more in-depth study of *E. chaffeensis* epidemiology in the area is required. This study represents the first direct evidence of a putative transmission cycle of this agent in Argentina with critical implications within a One Health framework. The convergence of threatened native fauna, generalist tick species, and expanding human activity in shared landscapes generate conditions conducive to pathogen emergence and spillover.

## Conclusions

The detection of *E. chaffeensis*, the etiological agent of human monocytic ehrlichiosis in a biodiverse wetland ecosystem increasingly exposed to anthropogenic pressure, highlights the need of being aware of potential tick borne *Ehrlichia* species. This underscores the urgency of implementing integrated, multidisciplinary surveillance systems that monitor environmental, animal, and human health as interconnected components. Such an approach is particularly relevant in ecotonal regions, where ecological disturbance, biodiversity conservation, and public health risks converge.

## Supplementary Information

Additional file 1. **Figure S1.** *Amblyomma triste* tick. (a) Dorsal view of an adult male specimen, (b) ventral view of an adult male, (c) ventral cuticle released from an adult male, and (d) salivary glands extracted from an adult male

Additional file 2. **Figure S2.** Phylogeny for *16S rRNA* gene based on 315 bp. Maximum likelihood tree. Substitution model: Kimura two-parameter model with discrete gamma distribution. Phylogeny was tested with 1000 replications of bootstrap. Sequences corresponding to the samples under study (CP_506, CP_508 and SG.At_508) are labeled with blue fonts. iTOL.v7 program was used for tree visualization

Additional file 3. **Figure S3.** Phylogeny for *groEL* gene based on 307 bp. Maximum likelihood tree. Substitution model: Kimura two-parameter model with discrete gamma distribution. Phylogeny was tested with 1000 replications of bootstrap. Sequences corresponding to the samples under study (CP_506, CP_508 and SG.At_508) are labeled with green font (bold font was used for the tick sample). Accession numbers from other *Ehrlichia* sp. found in tick samples are labeled with bold font. iTOL.v7 program was used for tree visualization

## Data Availability

All sequences obtained in the present study have been deposited in GenBank database under the following accession numbers: PV785953, PV786002, PV861766, PV791126, PV785954, PV786003, PV868190, PV791127, PV785955, PV786004, PV861764, and PV861765.
