# Supplementary figures and images for: Molecular detection of Ehrlichia chaffeensis in marsh deer (Blastocerus dichotomus) and their parasitic Amblyomma triste ticks in Argentina suggests a local transmission cycle
Source: Parasit Vectors. 2026 Jan 2;19:63. doi: 10.1186/s13071-025-07211-1 (PMC12866569; doi:10.1186/s13071-025-07211-1)

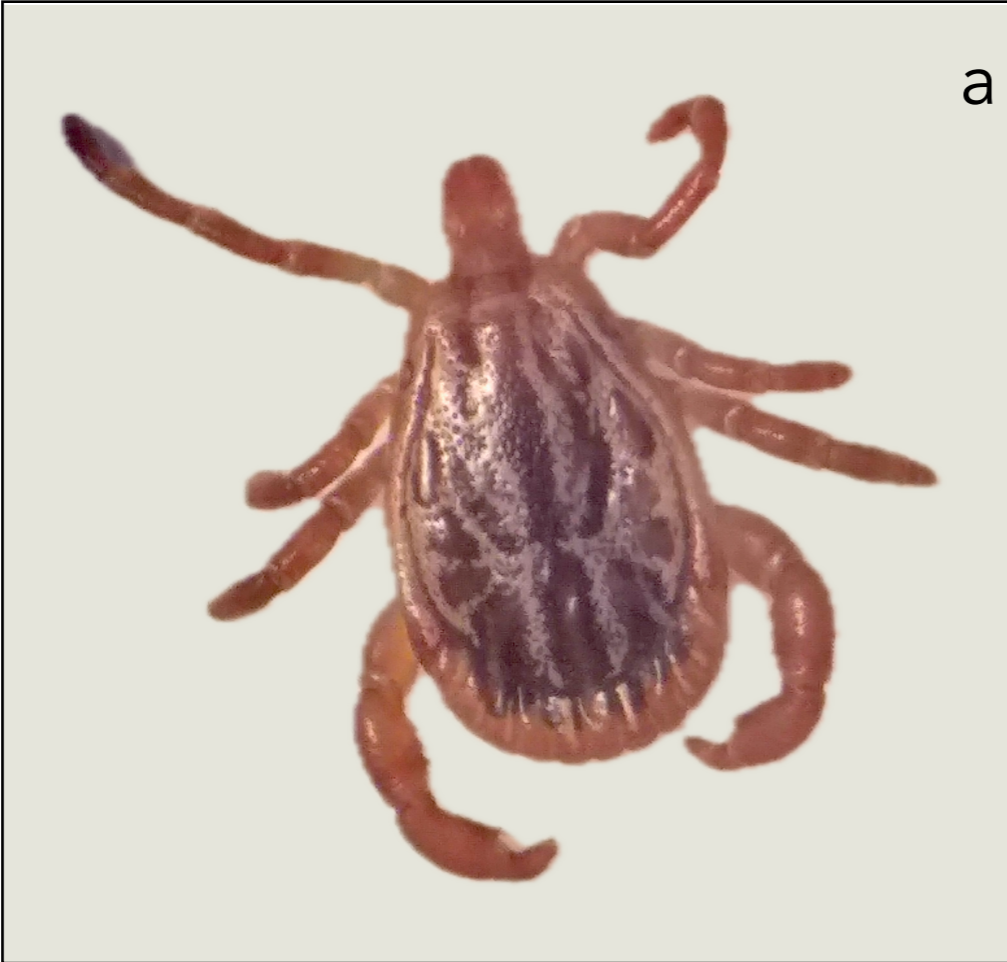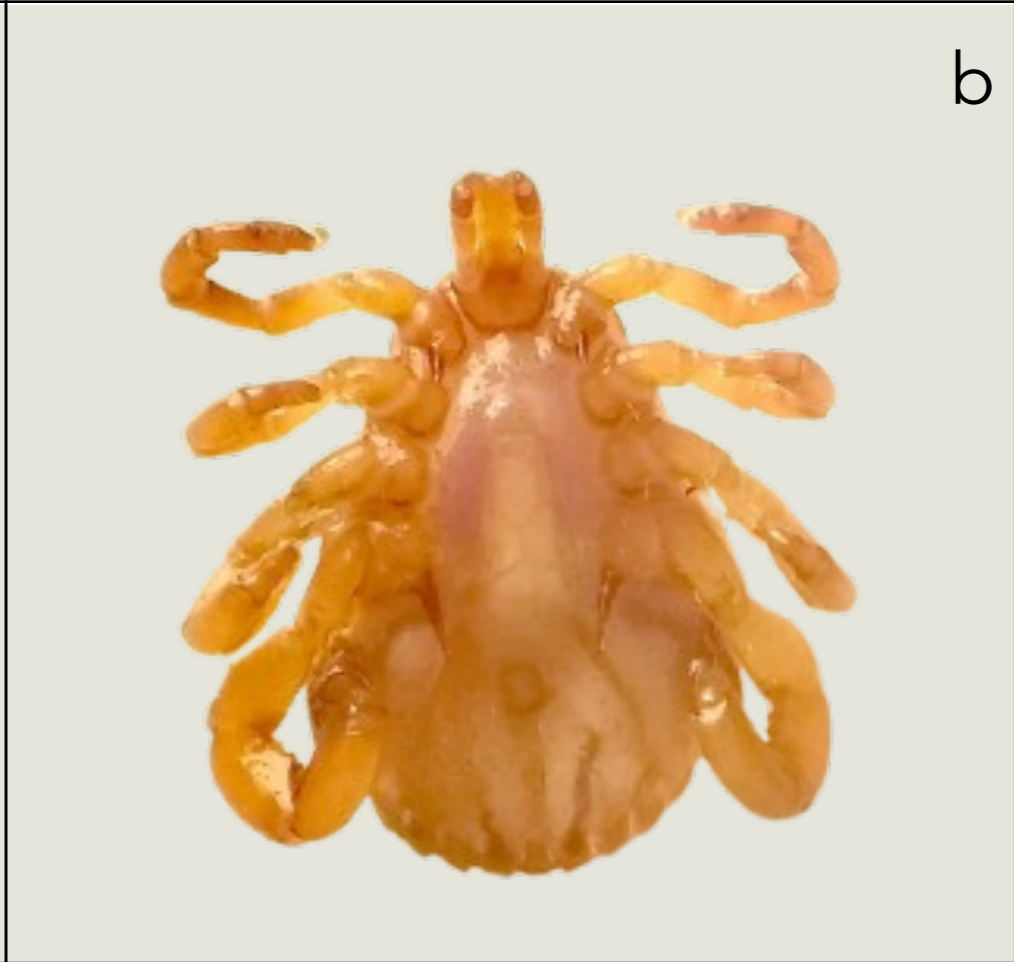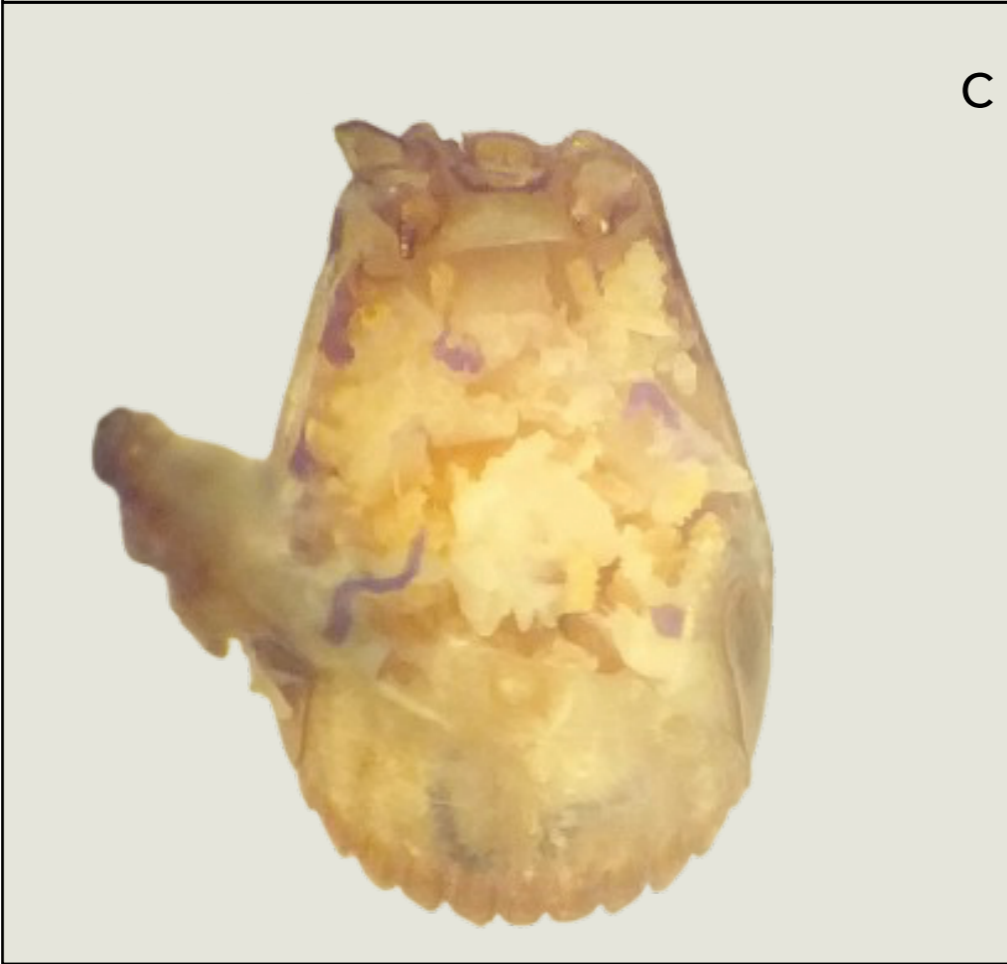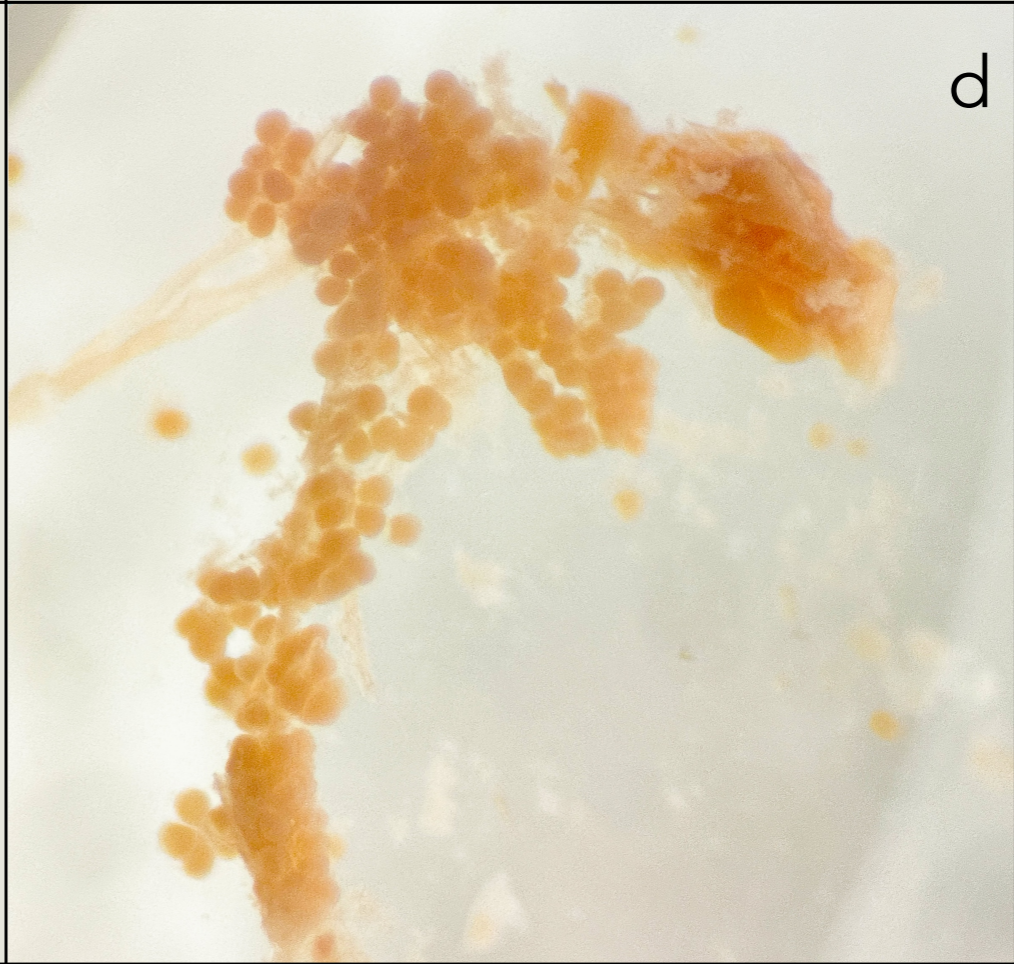

Supplement: Supplementary file 1 — Additional file 1. Figure S1. Amblyomma triste tick. (a) Dorsal view of an adult male specimen, (b) ventral view of an adult male, (c) ventral cuticle released from an adult male, and (d) salivary glands extracted from an adult male [file 13071_2025_7211_MOESM1_ESM.pdf]

Tree scale: 0.1

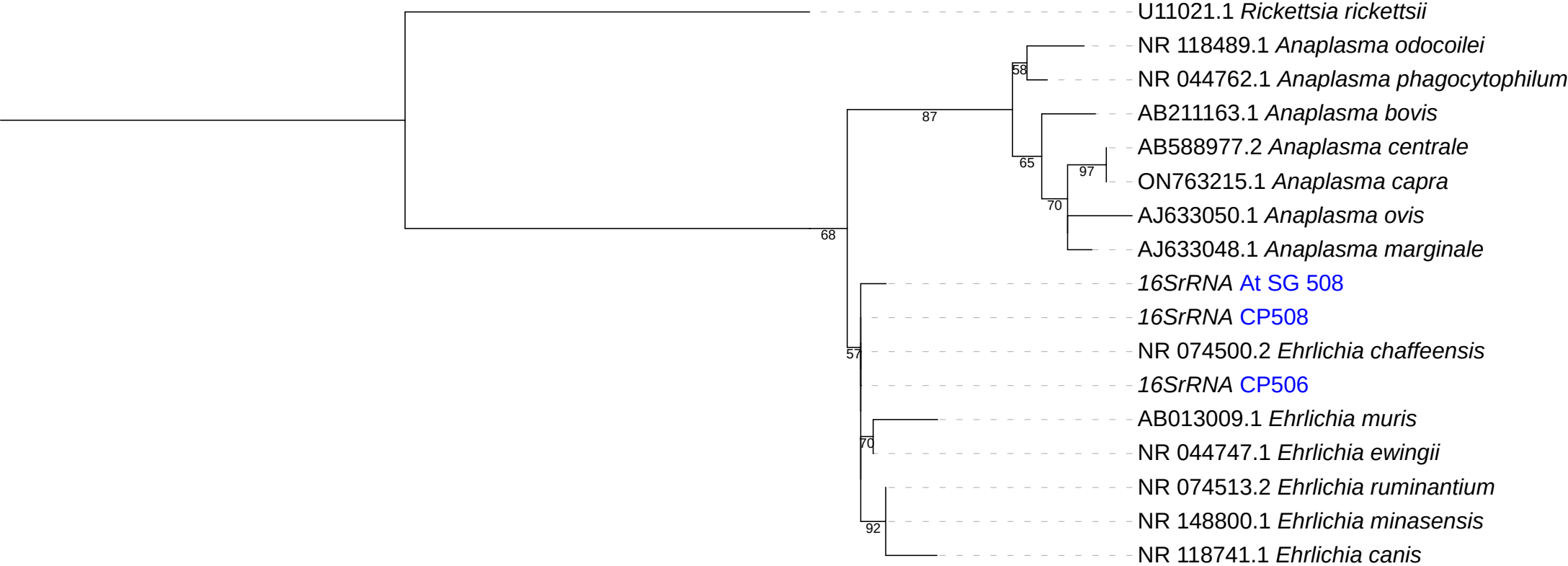

Supplement: Supplementary file 2 — Additional file 2. Figure S2. Phylogeny for 16S rRNA gene based on 315 bp. Maximum likelihood tree. Substitution model: Kimura two-parameter model with discrete gamma distribution. Phylogeny was tested with 1000 replications of bootstrap. Sequences corresponding to the samples under study (CP_506, CP_508 and SG.At_508) are labeled with blue fonts. iTOL.v7 program was used for tree visualization [file 13071_2025_7211_MOESM2_ESM.pdf]

Tree scale: 1

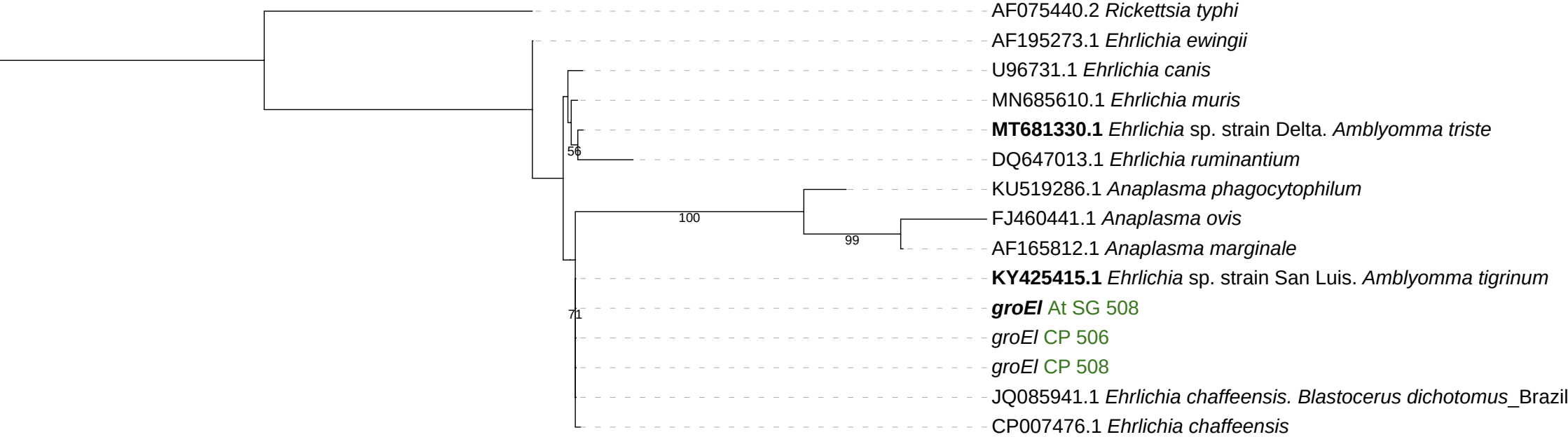

Supplement: Supplementary file 3 — Additional file 3. Figure S3. Phylogeny for groEL gene based on 307 bp. Maximum likelihood tree. Substitution model: Kimura two-parameter model with discrete gamma distribution. Phylogeny was tested with 1000 replications of bootstrap. Sequences corresponding to the samples under study (CP_506, CP_508 and SG.At_508) are labeled with green font (bold font was used for the tick sample). Accession numbers from other Ehrlichia sp. found in tick samples are labeled with bold font. iTOL.v7 program was used for tree visualization [file 13071_2025_7211_MOESM3_ESM.pdf]
